# Individuals living in a malaria-endemic area of Cameroon do not have an acquired antibody response to *Plasmodium falciparum* histidine-rich protein 2

**DOI:** 10.1186/s12936-017-1704-4

**Published:** 2017-02-01

**Authors:** Diane Wallace Taylor, Naveen Bobbili, Vedbar S. Khadka, Isabella A. Quakyi, Rose G. F. Leke

**Affiliations:** 1 Department of Tropical Medicine, Medical Microbiology and Pharmacology, John A. Burns School of Medicine, University of Hawaii at Manoa, 561 Ilalo Street, Honolulu, HI 96813 USA; 2 Office of Biostatistics & Quantitative Health Sciences, John A. Burns School of Medicine, University of Hawaii at Manoa, 561 Ilalo Street, Honolulu, HI 96813 USA; 3 Department of Biological, Environmental and Occupational Health Sciences, School of Public Health, College of Health Sciences, University of Ghana, Legon, P.O. Box LG 13, Accra, Ghana; 4 Faculty of Medicine and Biomedical Sciences, Biotechnology Center, University of Yaoundé I, Yaoundé, Cameroon

**Keywords:** *Plasmodium falciparum*, Malaria, Diagnosis, Rapid diagnostic tests, Histidine-rich protein II, HRP2, HRP-II, Antibodies

## Abstract

**Background:**

Diagnosis of *Plasmodium falciparum* is often based on detection of histidine-rich protein 2 (HRP2) in blood. Most HRP2-based assays have high sensitivity and specificity; however, authors have suggested that antibodies (Ab) to HRP2 could reduce assay sensitivity. This study sought to characterize the antibody response to HRP2 with respect to prevalence, class, subclass, affinity, and age distribution in Cameroonian children and adults residing in an area with high *P. falciparum* transmission.

**Methods:**

Plasma samples from 181 Cameroonian children and adults who had been repeatedly exposed to *P. falciparum* and 112 samples from American adults who had never been exposed were tested for IgG Ab to HRP2. For comparison, Ab to the merozoite antigens MSP1, MSP2, MSP3 and the pregnancy-associated antigen VAR2CSA were measured using a multiplex bead-based assay. In addition, 81 plasma samples from slide-positive individuals were screened for IgM Ab to HRP2.

**Results:**

As expected, children and adults had IgG Ab to MSP1, MSP2 and MSP3, antibody levels increased with age, and only women of child-bearing age had Ab to VAR2CSA; however, no convincing evidence was found that these individuals had an acquired antibody response to HRP2. That is, using two sources of recombinant HRP2, identical results were obtained when plasma from 110 Cameroonian adults and 112 US adults were screened for IgG Ab. Further studies showed that antibody prevalence and levels did not increase with age in Cameroonians between ages 5 and >80 years. Although a few samples from slide-positive Cameroonians had IgM values slightly above the American cut-off, it was unclear if the individuals had a true IgM response to HRP2 or if the values were due to non-specific binding from elevated immunoglobulin levels associated with infection. Data from prediction models showed a paucity of Class II T cell epitopes in HRP2.

**Conclusions:**

These data support the conclusion that most individuals in malaria-endemic areas do not produce an acquired humoral response to HRP2. The absence of Ab helps explain why HRP2-based assays are able to detect nanogram amounts of HRP2 and why HRP2 continues to circulate for a long time after parasite clearance.

## Background

In 1990, histidine-rich protein 2 (HRP2) was identified as a potential target for diagnosis of *Plasmodium falciparum* infections [1]. Soon thereafter, the approach for diagnosing malaria by detecting HRP2 in blood was validated when a field trial in Thailand (1993) reported that an antigen-capture ELISA for HRP2 [2] had 98% sensitivity and 96.2% specificity [3]. Since then, numerous rapid diagnostic immuno-chromatographic tests (RDT) for detecting HRP2 have been developed and used worldwide as an alternative to microscopy for diagnosing malaria. The WHO initiative launched in 2012 for control of malaria, T3: Test, Treat, Track, relies heavily on the use of RDT for diagnosis of malaria [4]. In addition, recent studies have measured the amount of HRP2 in blood as an index of whole-body parasite burden [5, 6] and to estimate disease pathology [7]. HRP2 has proven to be a valuable protein for diagnosis of malaria since it is produced by ring and trophozoite-stage parasites and secreted into plasma [6, 8]. Because infected erythrocytes containing trophozoite-stage parasites sequester in deep vascular tissues, detection of soluble HRP2 in blood plasma allows for diagnosis of malaria in individuals who are slide-negative by microscopy.

Since rabbits and mice readily produce antibodies (Ab) when immunized with recombinant HRP2, it is assumed HRP2 is immunogenic in people infected with *P. falciparum*. Accordingly, a number of authors have speculated that Ab to HRP2 might reduce the sensitivity of HRP2-based assays [3, 9, 10], since Ab could complex with soluble or parasite-associated HRP2 and block its detection in antigen-capture assays. Surprisingly, few groups have studied Ab to HRP2 [9–11]. In a drug-treatment study in India, Biswas et al. reported that soluble HRP2 and IgM Ab to HRP2 were present in *P. falciparum*-infected patients at enrolment, that both HRP2 and IgM to HRP2 declined following drug treatment, and that anti-HRP2 IgG Ab were present on days 15 and 28 days after drug treatment [10]. However, in a brief report, Das et al. tested plasma by ELISA from individuals living in a malaria-endemic region of India for Ab to HRP2 and found no difference in optical density (OD) values between 15 naive and 45 exposed individuals [11]; however, the lack of a positive control for the assay complicated interpretation of the results. More recently, Ho et al. using a similar ELISA reported that 25% of plasma samples (n = 75) collected from patients with acute malaria in Cambodia, Nigeria and the Philippines had anti-HRP2 Ab [9], with the majority of samples having OD values only one to three times above the cut-off. These results suggest that HRP2 is either not highly immunogenic in humans or the Ab response is short-lived.

Because of the potential for anti-HRP2 Ab to reduce the sensitivity of HRP2-based diagnostic assays, this study sought to establish the prevalence, class, subclass, affinity, and age distribution of Ab to HRP-2 in Cameroonian children and adults, aged 5 to 82  years of age, residing in a malaria-endemic region with high *P. falciparum* transmission. For comparison, Ab to the merozoite antigens MSP1, MSP2, MSP3 and the pregnancy-associated antigen VAR2CSA were measured using a bead-based multiplex assay. The study design tested the hypothesis that if Ab to HRP2 were produced by an antigen-specific acquired humoral immune response, then Ab would be present in individuals who had been exposed but not in unexposed individuals, and that the Ab response would be boosted by re-exposure. Plasma samples from 181 Cameroonian children and adults (45% of whom were slide-positive from malaria) were tested and compared with data from 112 Americans who had not been exposed to malaria. Overall, results for MSP1, MSP2, MSP3 and VAR2CSA were as expected; however, there was no solid evidence that individuals living in a malaria-endemic area had developed an acquired humoral immune response to HRP2.

## Methods

### Study design, plasma samples and clinical information

The first study compared Ab levels to HRP2, MSP1, MSP2, MSP3, and VAR2CSA between 110 Cameroonian and 112 American adults. Cameroon plasma samples were collected as part of a cross-sectional study between 1994 and 1999 and included subjects 18 and 80 years of age residing in Simbok, where residents received an estimated 566 infectious bites per year at the time of collection [12]. The USA plasma samples were acquired over the last 20 years from individuals residing in the USA, including plasma purchased from commercial blood suppliers (e.g., Interstate Blood Bank), blood donors for in vitro cultures of *P. falciparum*, discarded coded sample from clinical laboratories, and adults, including pregnant women, residing in Hawaii. In the study evaluating acquisition of Ab with age, children between the ages of 5 and <18 years were included, providing a panel of 132 individuals. The panel included: 5 to 10 years old (n = 9), 10–5 years (n = 12); 16–20 years (n = 8); 21–25 (n = 15) years; 26–30 years (n = 19); 31–40 years (n = 29); 41–50 years (n = 13); and >50 years (n = 27). To determine if active *P. falciparum* infection induced IgM Ab, plasma from 81 individuals (49 children and 32 adults) who were slide-positive for malaria were tested. Overall, plasma from 181 Cameroonian and 112 US individuals were screened for Ab to HRP2.

### Antigens

Two recombinant HRP2 proteins were used: recombinant HRP2-(A) was kindly provided by B. Pasloske (M-HRP2 4.22.92; Affymax Research Institute, Palo Alto, CA, USA) and recombinant HRP2 (B) was provided by D. Sullivan (Johns Hopkins University) [13]. For comparison, four additional malarial antigens were used, including a recombinant protein from the C-terminal regions of the merozoite surface protein 1 (MSP1-42) and MSP-2 produced in *Escherichia coli* provided by C. Long (Malaria Vaccine Development Branch, NIAID, NIH, USA); the C-terminal region of MSP3 expressed in *E. coli* (m.w. 22,000) from the Pasteur Institute; and, full-length recombinant VAR2CSA (FV2) produced in baculovirus provided by A. Salanti, University of Copenhagen. VAR2CSA was used, even though Ab to it are pregnancy-associated, because Americans have higher background levels to this protein than to other recombinant malarial antigens.

### The multiplex analyte platform for antibodies

**Fig. 1:**
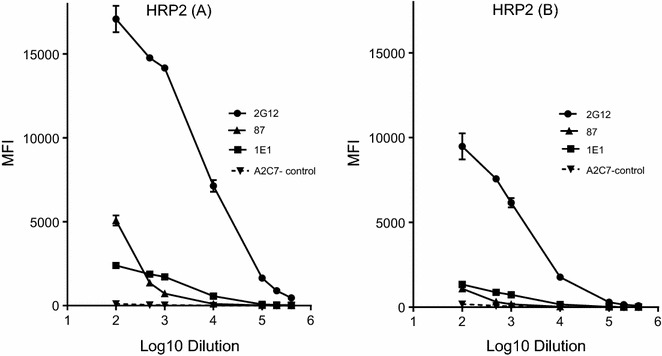
Titration of monoclonal antibodies to recombinant HRP2. Tenfold serial dilutions of murine ascites fluid containing monoclonal Ab to HRP2 (2G12, 87 and 1E1) and an irrelevant mAb (A2C7—negative control) were assayed on HRP2-coupled microspheres. Results are the mean ± SD for triplicate samples (technical replicates). The amount of HRP2-B coupled to the microspheres was fourfold lower than HRP2-A, explaining the difference in MFI values between the two datasets. The MFI observed with 1E1 (IgM) might be low, because the secondary reagent only detected IgM by light-chain reactivity

Malarial antigens were coupled to SeroMap microspheres as previously described [14]. To determine the optimal amount of HRP2 to use, 1, 2, 4, 8, and 16 µg of recombinant HRP2-(A) and HRP2-(B) were coupled to 10^6^ SeroMap microspheres. Essentially identical results were obtained with both recombinant proteins when coupled at equivalent concentrations. In the current study, beads were coupled with 4 µg of HRP2-(A) (optimal) but only 1 µg of HRP2-(B) per 10^6^ microspheres as the available supply was limiting. The lower values for HRP-(B), (e.g., Fig. 1) are due to the lower amount of protein coupled, not to protein quality. The malarial antigens MSP1-42, MSP-2 and MSP-3 were coupled at 1 μg/10^6^ microspheres and full-length VAR2CSA at 3 µg/10^6^ microspheres. In optimizing the assay, all antigen-coupled microspheres were tested against pools of positive and negative control plasma in the monoplex and multiplex format. No difference in results was obtained when the antigens were used alone or combined. In the assay, 50 μl of plasma at a 1:100 dilution in PBS + 1% BSA (unless otherwise specified) was incubated with 50 μl of the microsphere mix (pool of 2000 microspheres coupled to each antigen) at RT for 60 min on a shaker at 600 rpm. Microspheres were washed twice with 200 μl of PBS-0.05% Tween and then once with 200 μl of PBS + 1% BSA per well. The microspheres were resuspended and incubated with 100 μl of either: (1) anti-human IgG coupled to Phycoerythrin diluted in PBS + 1% BSA at 2 µg/ml (R-Phycoerythrin AffiniPure F(ab’) Fragment Goat Anti-Human IgG, Fcγ Fragment Specific, Jackson ImmunoResearch Lab, Inc., Cat# 109-116-170); or, (2) or anti-human IgM-phycoerythrin (R-Phycoerythrin AffiniPure F(ab’) Fragment Donkey Anti-Human IgM, Fc_5μ_ Fragment Specific, Jackson ImmunoResearch Lab, Inc., Cat# 709-116-073). After incubation in the dark at room temperature (RT) for 60 min on a shaker, microspheres were washed and resuspended in 100 μl of PBS + 1% BSA per well and read in a Liquichip L100 reader (Qiagen, Valencia, CA, USA) programmed to read at least 100 beads per antigen. Results are expressed as median fluorescent intensity (MFI). All experiments were repeated at least twice using two different sets of antigen-coated beads.

### Monoclonal antibodies (mAb) to HRP

Three mAb to HRP2 (mAb 87, mAb 2G12-1C12, and mAb 1E1-A9) and an irrelevant mAb (A2C7-A3B11) were used. Previous studies showed that mouse mAb87 (IgM) detects HRP2 by metabolic immunoprecipitation and western blotting [15]; 2G12 (IgG) binds to the sequence DAHHAADAHH in the repeat region of HRP2 and 1E1 (IgM) binds to AHHAHHV [16, 17]. MAb 2G12 and 1E1 work especially well together in an HRP2-based diagnostic ELISA [2]. Sequencing studies showed that epitopes recognized by 2G12 and 1E1 are present in 100% (448/488) and 99.3% (445/448) of *P. falciparum* isolates, respectively [17]. The mAb were used in this study to verify that these two major epitopes of HRP2 that are present in multiple strains were correctly displayed on the coupled microspheres. Serial dilutions of the anti-HRP-2 and control mAb were incubated with the HRP-2 coupled microspheres as described above. Following incubation, the microspheres were incubated with 100 μl of R Phycoerythrin-coupled AffiniPure F(ab’) Fragment Donkey Anti-Mouse IgG (H + L) (Jackson ImmunoResearch Lab, Inc., Cat# 715-116-150) diluted in PBS + 1% BSA at 2 µg/ml.

### Per cent of high avidity antibody to HRP2

The avidity assay was performed as previously described with minor modifications [18]. Briefly, plasma was diluted 1:300 in 1% BSA-PBS and 50 µl was added to duplicate wells containing 50 µl of antigen-coupled microspheres (2000 microspheres/test) and incubated for 1 h on a shaker. After incubation, 100 µl of 3 M NH_4_SCN in 1% BSA-PBS was added to half the wells and 100 µl of 1% BSA-PBS was added to the other half. After 30-min incubation, the wells were washed and incubated with R-phycoerythrin-labelled anti-human IgG, washed, and analysed by Liquichip L100 as described above. Avidity was calculated by (MFI obtained from wells incubated with salt)/(MFI obtained from corresponding control wells) × 100 for each antigen. Positive and negative controls were included on each plate.

### Indirect immunofluorescent assay (IFA)

*Plasmodium falciparum* (FVO) were cultured in vitro until parasitaemia reached ~5 to 10% and blood smears were prepared. Following fixation in cold acetone, selected plasma samples were diluted 1:50 and ~15 μl was applied to the smears for 30 min at RT. Slides were washed with PBS and incubated with 15 μl of a 1:100 dilution of fluorescein isothiocynate (FITC)-labelled anti-human IgG (AffiniPure F(ab’) Fragment Goat Anti-Human IgG, Fcγ Fragment Specific, Jackson ImmunoResearch Lab, Inc., Cat# 109-096-098) or anti-human IgM (rabbit anti-human IgM (Fc specific, Sigma, Cat # 14121 followed by FITC-AffiniPure F(ab’) Fragment Goat Anti-Rabbit IgG (H + L), Jackson ImmunoResearch Lab, Inc., Cat# 111-096-003). MAb 2G12 and IE1 and normal mouse plasma were used as a positive and negative controls, respectively, because the MAb give a distinct pattern of fluorescence when bound to HRP2. After 30 min, smears were washed, mounted in mounting medium (Barbital buffer, pH 8.5 in 50% glycerol), and examined using a Zeiss Axiovision HBO 100 fluorescent microscope.

### Algorithms for detecting potential Class II T cell epitopes

Based on information in the original sequencing study [19], *P. falciparum* NF135/5.C10 GenBank: ETW43476.1 was used in the analysis and included the 28 amino acids prior to the repeat sequence, the repeat sequence (R_II_), and 9 amino acid C-terminal region. The sequence was analysed for MHCII binding predictions using the IEDB analysis resource Consensus tool based on data for 4/17/2-13 [20, 21]. The predicted outputs are given as IC 50 nM binding for the combinatorial library and SMM_align; with scores <50 nM considered to be high, <500 nM intermediate affinity, and <5000 nM as low affinity binding. Allelic/frequency distributions for Africa were obtained from the Allelic Frequency Net Database in Worldwide Populations [22].

### Statistical analysis

Comparisons of MFI between different groups are presented as mean ± SD. Cut-off for sero-positivity was based either on mean ±3 SD or area under the receiver operator curves (ROC), as specified in the text.

### Ethical approval

Coded, archival plasma samples and clinical information were used in this study. It received exemption from the Committee on Human Subjects, University of Hawaii (CHS #23048). The Cameroonian samples and data were originally collected with approval of the University of Yaoundé 1 Ethics Committee, Georgetown University (IRB #1994-158), and Office of Human Research Protection, Department of Health and Human Services(single project assurance #S-9601-01).

## Results

### Assessing if recombinant HRP2 was appropriately coupled to the microspheres

HRP2-coupled microspheres were incubated with serial dilutions of 3 mAb to HRP2 and control mAb (Fig. 1). Based on a cut-off of the mean plus 3 standard deviations (SD) of the irrelevant mAb control, mAb 2G12 (IgG) had a titre of >1:100,000 and mAb 87 and 1E1 had titres of >1:1000 on both recombinant HRP2 proteins. The relationship between MFI and log Ab dilution was linear between 500 and 18,000 MFI, demonstrating a direct relationship between antibody concentration and MFI. These results confirmed that epitopes important in HRP2-based RDT are correctly displayed on the microspheres.

### Titration of pooled human plasma

**Fig. 2:**
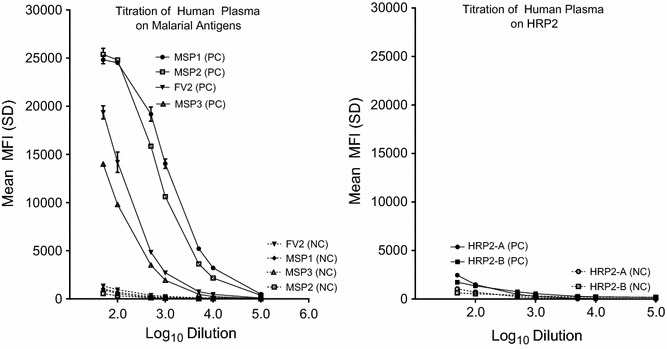
Titration of pooled positive and negative plasma on malarial antigens. Tenfold serial dilutions of pooled plasma from 11 Cameroon adults (PC) and a pool of plasma from 11 non-malarial exposed Americans (NC) were assayed for IgG Ab to MSP1, MSP2, MSP3, FV2 and 2 recombinant preparations of HRP2 (A and B). Results are the mean ± SD for triplicate wells. Cut-off for positivity (mean + 3 SD) ranged from 500 to 1350 MFI at 1:50 dilution. *PC* positive control (*solid lines*) and *NC* negative control (*dotted lines*)

For this study, plasma from 11 Cameroonian adults >18 years of age residing in Simbok were randomly selected and combined in equal volumes to create a positive control (PC) and samples from 11 American adults were randomly selected and pooled to produce a negative control (NC). The pooled PC and NC controls were serially diluted and tested against the malarial antigens (Fig. 2). As expected, the PC had high titres of IgG for MSP1, MSP2, MSP3, and FV2, whereas, the NC did not. Surprisingly, no difference was seen between the PC and NC for HRP2. Therefore, the PC pool from Cameroon gave a robust response to the four *P. falciparum* antigens, but not to HRP2.

### Comparison of IgG levels in individual Cameroonian and Americans

**Fig. 3:**
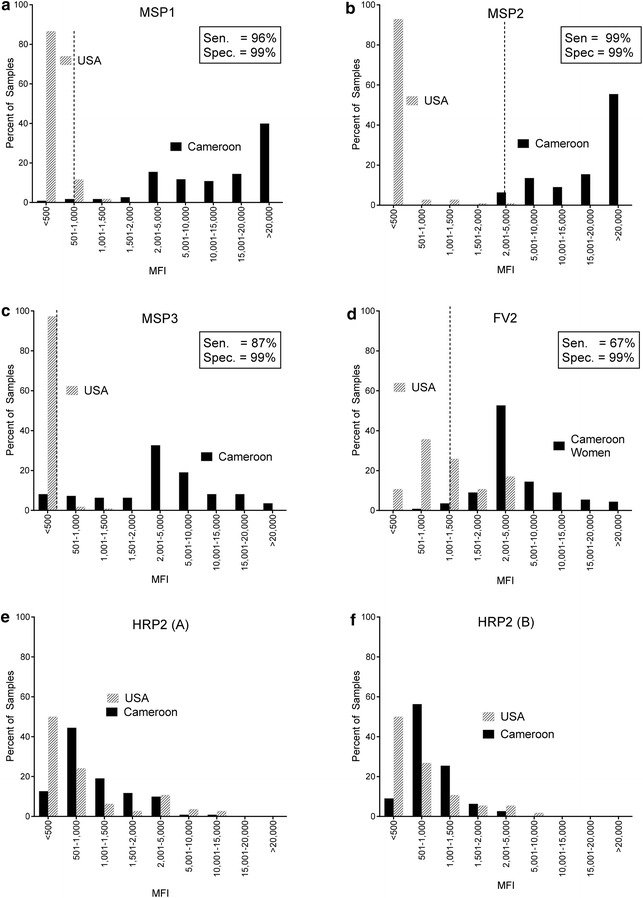
Distribution of MFI for Cameroonian and American adults. MFI values for each antigen for 110 Cameroon and 112 American adults were divided into 9 bins of unequal sizes, in order to expand the lower part of the distribution. The ROC was used to determine optimal cut-offs (*vertical dotted lines*) for the best overall sensitivity and specificity. Cut-offs: **a **MSP1-42 = 793 MFI; **b **MSP2 = 2889 MFI; **c **MSP3 = 244 MFI; **d **FV2 = 1345 MFI, **e** and **f** no ROC cut‐off

Since the PC contained samples from only 11 Cameroonian adults, it was possible that only a small fraction of individuals had Ab to HRP2. Therefore, plasma samples from 110 Cameroonians (≥18 years of age) and 112 Americans were screened individually for IgG Ab against the antigens. The frequency distribution of MFI in the samples when tested at a 1:100 dilution is shown in Fig. 3. As expected, MFI were low (<1000) in Americans for MSP, MSP2 and MSP3 and fewer than 5000 MFI to FV2. Using ROC analysis, it was easy to establish a cut-off providing >85% sensitivity and 99% specificity (Fig. 3a–d). However, for HRP2, there was no difference in the frequency distribution of MFI between the Cameroon and US samples. In fact, the highest MFI were actually found in the US samples (Fig. 3e, f).

### Measuring HRP2 antibody levels with increasing age

**Fig. 4:**
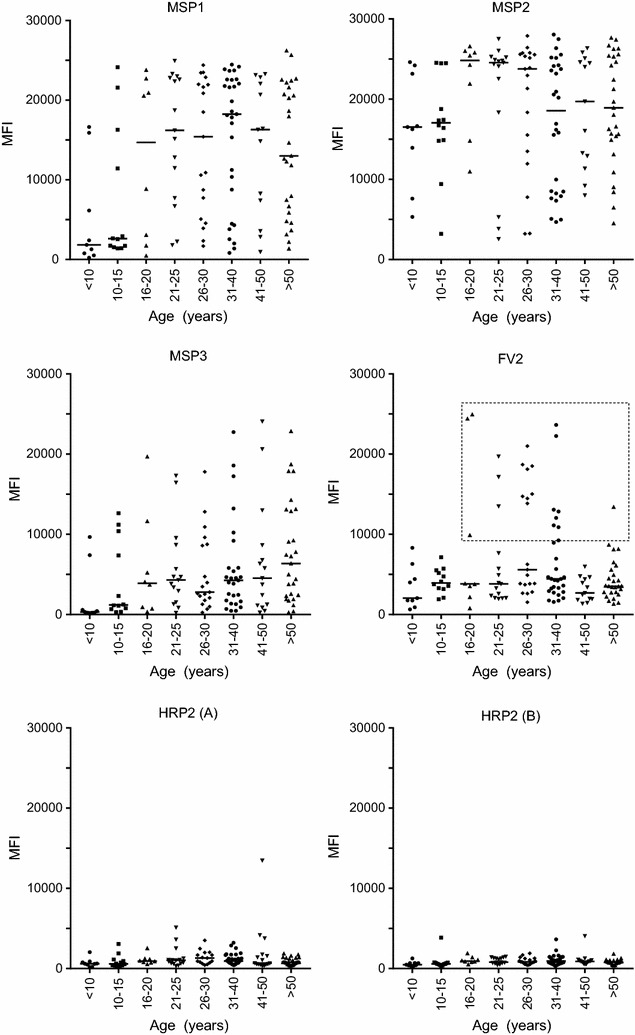
Change in antibody levels with age. Ab levels were measured for 132 individuals aged 5 to 80+ years.* Horizontal lines* indicate the median for each group. Since only pregnant women produce Ab to FV2, only women over 16 years of age had Ab to this antigen (Ab positive women are located within the square). (Sample size: n = 9 individuals, <10 years; n = 12, 10–15 years; n = 8, 16–20 years; n = 19, 26–30 years; n = 29, 31–40 years; n = 13, 41–50 years; n = 27, >50 years)

One hallmark of an acquired IgG response is an increase in Ab levels with repeated exposure to the antigen. Accordingly, antibody levels to HRP2 and the other four antigens were measured in plasma samples collected from individuals between the ages of 5 and 80+ years residing in the peri-urban village (Fig. 4). As expected, Ab levels to MSP1, MSP2 and MSP3 increased with age, reaching maximal levels by age 16–20 years, and Ab to FV2 were only detected in women of child-bearing age (square in Fig. 4). However, antibody levels remained low to the HRP2 proteins, without a significant age-related increase.

### Comparison of IgM levels in malaria slide-positive individuals and US controls

**Fig. 5:**
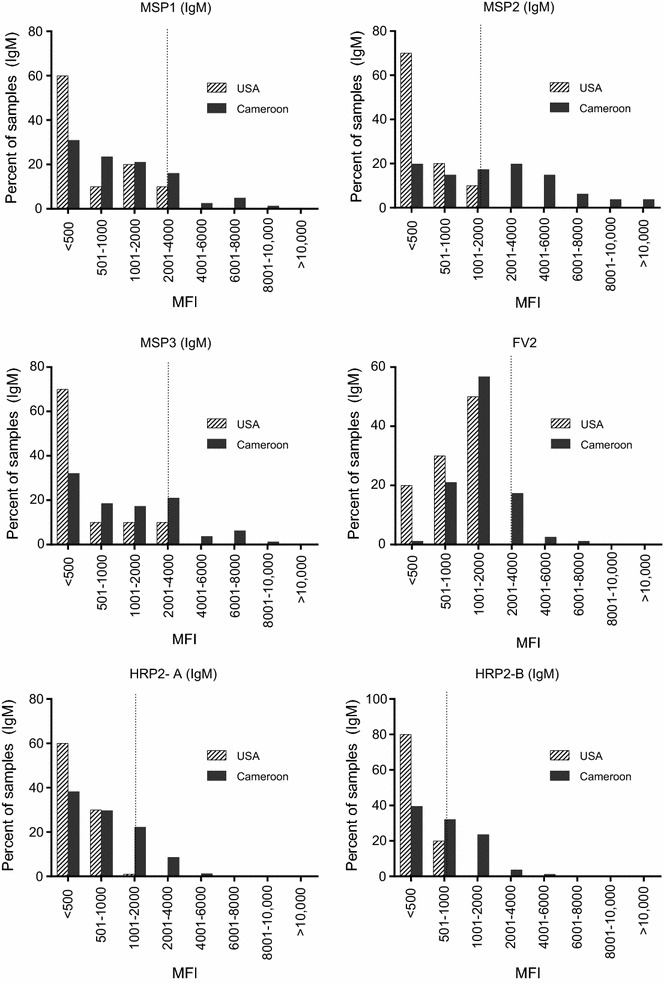
IgM levels to malarial antigens. Plasma from 81 individuals who were blood-smear positive for *P. falciparum* were screened for IgM Ab to the antigens shown. The *dotted vertical line* identifies the centre of the bin where the value for the mean + 3 SD for US control samples is located

Although IgG Ab were not detected, HRP2 might induce a T-independent IgM response since the protein has multiple tandem repeat sequences. Because IgM has a short half-life, 81 plasma samples from children (n = 49) and adults (n = 32) who were slide-positive of *P. falciparum* were screened for IgM Ab, as well as ten randomly selected US controls (Fig. 5). US controls had low reactivity for IgM Ab to HRP2-(A) (mean 442 MFI) and HRP2-(B) (369 MFI). Unfortunately, the ROC method provided poor discriminatory ability for establishing a cut-off (e.g., sensitivity <20% with a specificity >97%). Using mean plus 3 SD of US controls samples, 11, 57 and 17% of malaria-infected Cameroonians had IgM Ab to MSP1, MSP2 and MSP3, respectively; 15 and 30% had IgM Ab to HRP2-A and HRP2-B, with the highest MFI values for these antigens reaching only 4930 and 3823 MFI. It is unclear if samples with values above cut-off actually: (1) contain Ab to HRP2; (2) were due to increased non-specific binding of polyclonal IgM (high background); or, (3) detected cross-reactive Ab.

### Testing IgG antibody avidity

**Fig. 6:**
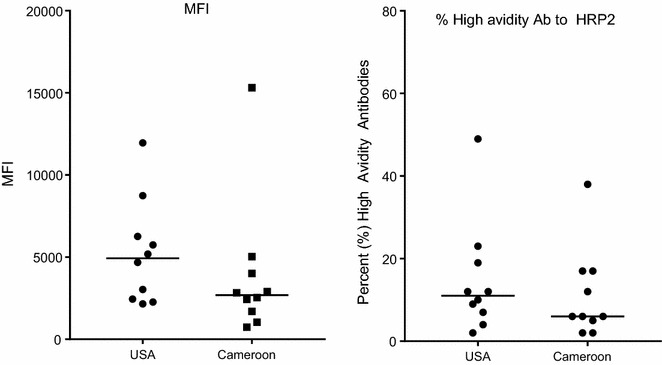
Testing the strength (avidity) of IgG binding in plasma the 10 Cameroonian and 10 USA adults with the highest MFI for HRP2-A. The 10 American and 10 Cameroonian plasma samples with the highest MFI for HRP2 were incubated with 1% BSA-PBS and 3 M NH4SCN in 1% BSA-PBS and the percentage of Ab that remained bound was determined. Results showed that at 1:100 dilution all plasma samples had consistently low level binding to HRP2 and there was no difference in the strength of interaction between plasma from Americans and Cameroonian adults. *Horizontal bar* median for the group

Since Ab secreted by antigen-specific B cells usually have higher avidity than cross-reactive Ab, the avidity to HRP2 was tested using the ten Cameroonian and ten American samples with the highest MFI to HRP2-(A) (Fig. 6). Ab levels in MFI to HRP2 are shown in Fig. 6a and avidity % remaining bound in Fig. 6b. Interestingly, median Ab levels and avidity values were slightly higher in Americans who had never been exposed to malaria compared to Cameroonian adults. There was no evidence that plasma samples with the highest MFI contained Ab that were more specific (i.e., high avidity) for HRP2 in malaria-exposed compared to malaria-naive individuals.

### Testing of US plasma samples for binding to *Plasmodium falciparum* parasites

**Fig. 7:**
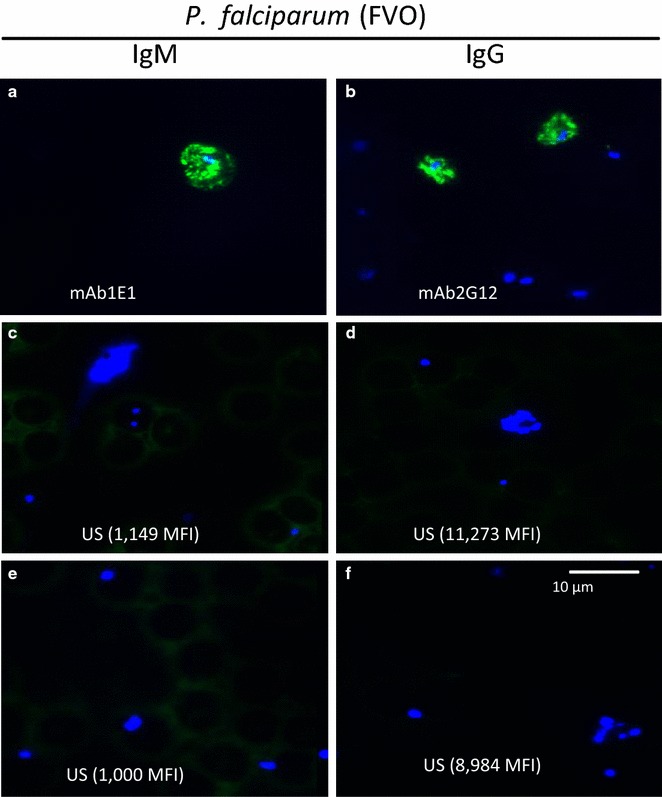
IFA staining of mAb to HRP2 and the four US samples with highest MFI for HRP2 **a**, **b** MAb 1E1 and 2G12 showed strong reactivity with the cytoplasm, transport vesicles within the erythrocyte cytosol, and at the surface of the infected erythrocyte. **c**–**f** In contrast, none of the four USA plasma samples (with the highest MFI in the multiplex analyte assay was positive (tested at 1:50 dilution). *Apple*–*green* Ab staining. Slides were counter-stained with DAPI to identify parasite nuclei (MFI values for the sample are shown in parentheses)

Since some American adults had IgG Ab that reacted with HRP2 (Fig. 3), it was possible they had Ab that cross-reacted with *P. falciparum* HRP2. Accordingly, samples from the six Americans with the highest MFI were tested by indirect immunofluorescent assay (IFA) for reactivity with *P. falciparum*-infected erythrocytes (Fig. 7). MAb 2G12 and 1E1 were used as positive controls as they produce a characteristic HRP2 staining pattern, including bright fluorescence in transport vesicles. None of the US samples had IgG or IgM Ab that bound to malaria infected-erythrocytes.

### Screening HRP2 for potential Class II T cell epitopes

Among the top 100 potential T cell epitopes predicted by consensus (including comb.lib/smm/nn methods), surprisingly only one high affinity association was found for any sequence within HRP2 and any HLA allele. The single high affinity interaction predicted by the Consensus method was between HLA-DRB1*11.01 and HRP2 amino acids 14–28 (core LNLNKRLLH), a sequence located N-terminal to the repeat sequence. Several other potential T epitopes with intermediate and weak interactions were predicted with the N-terminal region for binding with 11 other MHC Class II alleles. Within the repeat region (R_II_), no high affinity interactions were found; only one intermediate binding was predicted, which was between amino acids 45–113 with HLA-DQA1*05:01/DQB1*03:01 (mean IC_50_ value 108 nM) and one weak interaction between with HLA-DRB3*01:01 (mean IC_50_ of 3862 nM). No T cell epitopes were predicted at the C-terminus. A large number of HRP-2 sequences with high affinity binding for multiple Class II alleles was not found, suggesting HRP2 might not be able to provide adequate T cell help for generation of an acquired memory B cell response.

## Discussion

HRP2 is a unique protein, both in amino acid sequence and protein structure. It is 65–85 kDa protein made up of 35% histidine, 40% alanine and 12% aspartate [19, 23] that consists primarily of the repeat sequences AHH and AHHAAD [19, 23]. Once synthesized, HRP2 is transported via vesicle to the infected erythrocyte membrane, where [3H]-labelling studies show that galactose is attached either during, or just before, HRP2 is released into plasma [23]. Although the structure of HRP2 has been difficult to determine, data predict the repeat sequences form a 3/10 helix or a tightly coiled α-helix [23]. Thus, HRP2 is very different from known protein antigens.

HRP2 was initially selected for diagnosis of falciparum malaria because it is highly conserved, synthesized by ring-stage parasites, and secreted into plasma. Lee et al. sequenced 448 global isolates and reported that the sequences DAHHAAHAHH (recognized by mAb 2G12) and AHHAHHV (recognized by mAb 1E1) were present in 448/448 (100%) and 45/448 (99.3%) of the isolates, respectively, and that multiple copies of these repeat sequences were present (average 18.4 copies/isolate) [16, 17]. The binding of monoclonal Ab to HRP2-coupled microspheres demonstrates that epitopes in the repeat sequence were correctly displayed. Since individuals infected with *P. falciparum* are exposed to the repeat sequences of HRP2, they should have Ab to HRP2 if the protein is immunogenic.

The initial goal was to determine the prevalence, class, subclass, and avidity of Ab to HRP2 in different age groups living in a malaria-endemic region of Cameroon. The samples, collected between 1994 and 1999, were from a cross-sectional malaria prevalence study in Simbok village, at which time the estimated entomological inoculation rate was ~556 infectious bites annually [12]. Study subjects had been infected with *P. falciparum* multiple times. Based on the characteristic of acquired B cell responses, one would expect to find Ab to HRP2 in Cameroonian, but not American adults and that Ab prevalence, amount and avidity would increase with age. Interestingly, the results found no evidence of an acquired immune response to HRP2, since values for IgG to HRP2 were similar in exposed and unexposed individuals and IgG levels did not increase with age (Figs. 2, 3). The lack of IgG was not due to poor quality of the plasma samples, because the samples contained high levels of Ab to the other malarial antigens (MSP1, MSP2, MSP3) when tested simultaneously in the multiplex assay (Fig. 2). Because HRP2 has tandem repeats, it might function as a T-independent antigen. This possibility seemed unlikely since a related plasmodial parasite, *Plasmodium yoelii*, lacks both type 1 or 2 T-independent antigens, i.e., infected athymic mice fail to produce Ab to any malarial antigen [24]. The mice, however, had activated B-cells that did not secrete Ab in the absence of T cell help. Nonetheless, plasma from 81 Cameroonians who were blood-smear positive of *P. falciparum* were tested for IgM Ab to HRP2 (Fig. 4). A few individuals had borderline-positive IgM values for HRP2 compared to the US negative controls but MFI were very low. Since total IgM levels are elevated during *P. falciparum* infection due to antigen-specific and polyclonal B cell activation, it was difficult to know if values above the arbitrary cut-off were due to IgM Ab, non-specific binding, or cross-reactive Ab. Therefore, among the 112 American plasma samples, the six samples with the highest MFI for HRP2 were tested for IgG and IgM binding to *P. falciparum*-infected erythrocytes by IFA (Fig. 6). None of the US samples tested positive, suggesting that the Americans did not have Ab that cross-reacted with *P. falciparum*; rather, the samples simply had high backgrounds. The finding that antibody avidity was similar between Americans and Cameroonians for HRP2 (Fig. 6b) supports the conclusion that Cameroonians adults did not have an acquired immune response to HRP2, that is, a response that had undergone somatic hypermutation, affinity maturation and development of B cell memory.

Results from the current studies are similar to those reported previously. Using an IgG ELISA assay, Das et al. reported that OD of plasma samples were the same between malaria-naive individuals and those who had acute symptomatic infections, asymptomatic infections and uninfected adults residing in a malaria-endemic area in India [11]. Biswas et al. used a similar ELISA with a cut-off based on replicates of a pool of five negative plasma samples and reported detecting IgM Ab to HRP2 in patients at enrolment. However, following drug treatment, IgM Ab quickly declined and IgG Ab to HRP2 were detected 15–28 days post treatment. Patients in the above study had asymptomatic infections, showing they had sufficient immunity to control their infections. Yet they tested positive for IgM at enrolment and only produced IgG several weeks after drug treatment, thus displaying characteristics of a primary Ab response, suggesting they lacked a memory B cell response to HRP2 prior to the ongoing infection. Ho et al. used a similar ELISA and their ‘raw data’ looks similar to that found in Fig. 2 of the current study. The biggest difference between the two studies is the method used to establish the cut-off for positivity. Ho et al. used a pool of six samples from non-malaria exposed individuals as a negative control and converted OD into arbitrary units based on a pool of five samples with the highest OD to HRP2 [9]. They reported that 19/75 individuals with acute malaria in Cambodia, Nigeria and Philippines were Ab-positive and three/28 asymptomatic Solomon Island residents were positive. However, most positive samples had antibody levels only one to three times higher than the cut-off, with only two plasma samples having Ab levels fivefold and 16.7-fold above cut-off. In the current study, 112 malaria-naive control individuals were used, providing data for a wide range of malaria-naive individuals, including individuals with other infections (Fig. 2). Compared to MSP1, MSP2 and MSP3, a highly variable background level of reactivity was found for HRP2 in malaria-naive adults, with some individuals having 20-fold higher levels than others. Using plasma from a limited number of healthy controls to establish a cut-off for malaria-infected individuals with hyper-gammaglobinaemia may not be optimal. Overall, none of the above studies found high IgG antibody levels to HRP2 in people living in malaria-endemic regions. It is unlikely that IgG Ab to HRP2 significantly alter the sensitivity of HRP-2-based diagnostic assays.

The lack of Ab may help explain why HRP2-based assays work as well as they do. First, if Ab were present, soluble HRP2 would rapidly form immune complexes and quickly be cleared by the spleen. HRP2 is detectable in plasma for >28 days after parasite elimination, suggesting that the protein circulates freely in plasma and is slowly removed by glomerular filtration and proteolysis. Second, the detection thresholds in RDTs is very low, for example Marquat et al. reported that the lower limit of detection for four RDTs was between 6.9 and 27.8 ng/ml [25], an amount of antigen that would be neutralized by microgram levels of Ab. Finally, absence of Ab could explain why an association exists between parasite burden and disease severity, i.e., the more parasites, the more HRP2 in circulation. The uniqueness of HRP2 and its apparent poor immunogenicity would help explain why HRP2-based tests work the way they do.

Finally, the question is, why do people not produce Ab to HRP2? A number of possibilities exist. First, soluble proteins are usually poorly immunogenic in the absence of adjuvants. This explanation seems unlikely since HRP2 is also associated with the erythrocyte membrane. Secondly, endogenous histidine rich glycoprotein (HRG) exists in human plasma that also binds to divalent metal ions. However, Ab to HRG do not bind to HRP2 and mAb to HRP2 do not bind to HRG [23], making it unlikely humans are immunologically tolerant to HRP2. Third, Das et al. found that peripheral blood mononuclear cells from malaria-exposed adults proliferated and secreted IL-4 when stimulated in vitro with MSP1-42, but not with HRP2 [11]. They suggested that HRP2 might suppress some immune responses, including T cell proliferation, IFNɤ secretion and CD69 expression [26]. Fourth, HRP2 may lack an adequate number of T cell epitopes to induce an acquired B cell response in most individuals. Using predictive models, only one predictive Class II T cell epitope was identified, namely, the core sequence LNLNKRLLH located before the repeat sequences that only has high affinity for DRB1*011.01. Within the repeat region, which contains the majority of amino acids, only one T epitope with intermediate-binding was predicted, i.e., amino acids 45 to 113 with HLA-DQA1*05:01/DQB1*03:01 and one weak interaction with DRB3*01:01. A search of the Allele Frequency Net Database showed these alleles are not common in Africa, e.g., DRB1*11.01-DQA1*05:01 -DQB1*03:01 is present in 1.8–2.6% in Ethiopia and HLA-DRB3*01:01 was not reported. Since HRP2 has a unique amino acid composition and structure, it is plausible it may not be highly immunogenic in humans.

## Conclusions

These data support the conclusion that most individuals in malaria-endemic areas do not develop an acquired B cell immune response to HRP2. Lack of Ab to HRP2 help explain why HRP2-based assays are able to detect nanogram amounts of HRP2 and why soluble HRP2 continues to circulate for a long time after parasite clearance.

## Abbreviations

Ab: antibodies
FV2: full-length VAR2CSA (recombinant protein)
HRP2: *P. falciparum* histidine-rich protein 2 or HRP-II
HRG: histidine rich glycoprotein
IFA: indirect immunofluorescent assay
MFI: mean fluorescent intensity
MSP: merozoite surface protein
mAb: monoclonal antibody
OD: optical density
PC: positive control
RDT: rapid diagnostic test
ROC: area under the receiver operator curve
NC: negative control
